# Child Care and Carcerality: Reviewing Dorothy Roberts' “Torn Apart”

**DOI:** 10.1089/heq.2023.0119

**Published:** 2023-10-30

**Authors:** Marie-Fatima Hyacinthe

**Affiliations:** Department of Social and Behavioral Sciences, Yale University School of Public Health, New Haven, Connecticut, USA.

In “Torn Apart: How the Child Welfare System Destroys Black Families and How Abolition Can Build a Safer World,” Dorothy E. Roberts builds on years of research to create a thorough analysis of the US child welfare system. Roberts delivers a methodologically and theoretically rich book, which employs legal research, interviews, and media analysis to paint a vivid picture of how the child welfare system is both grounded in and furthers carceral systems. In keeping with the values of prison industrial complex (PIC) abolitionists, this book draws deeply from the experiences of advocates, activists, and those directly impacted by the child welfare system. Roberts offers multiple strategies for building a world that actually offers care for children without reinforcing racist, sexist, homophobic, transphobic, and classist logics.

Roberts melds historical context with interviews of child welfare workers, other academics, and activists. Her examples show how logics criminalizing poverty and enforcing colonial settler norms have legitimized a system that produces racial and class-based disparities and inequities in child removal and family surveillance. Roberts cites that Black children were 14% of the children in the United States in 2019, they represented 23% of the children in foster care nationally. These disparities become starker at the local level: In New York, Black children are 15% of the child population but 44% of the foster care population.

Roberts explains that the links between the child welfare system and the carceral system are fundamental; at the root is the urge to criminalize, pathologize, and stigmatize poor people, people of color, queer people, indigenous people, and others who do not fit white middle class norms. This analysis is facilitated by Roberts' engagement with communities that are directly affected by both systems, and their assessment of these structures. Roberts writes that the theory underlying this book was forged in the crux of movements to abolish the prison industrial complex and to strengthen families in the face of surveillance and policing. Thus, this book is not only a feat in research but is also exemplary of the kind of important scholarship that can take place in alliance with social justice movements.

Roberts also depicts how the current state of child welfare services was shaped by legislation that decreased funding for support and increased funding for policing, surveillance, and family separation. In 1996, Congress ended welfare by passing the Personal Responsibility and Work Opportunity Reconciliation Act. A year later, President Clinton signed the Adoption and Safe Families Act which incentivized sending children through foster care to be adopted rather than family reunification. Both of these laws passed in the aftermath of the 1994 Crime Law, creating an environment where the only “support” for families in danger is anchored in the carceral system.

Both the PIC and the child welfare system impinge on a person's right to “raise the children they have in dignity.”^1,2^ As in her previous works, Roberts demonstrates how this tenet of reproductive justice is degraded by a system that observes pregnant people, waiting to prove that they are a danger to their child.^3,4^ Roberts demonstrates that surveillance moves beyond hospital drug tests and into classrooms, pediatric appointments, and even other social service agencies. This creates an environment where poor families, those most likely to come into contact with these various surveillance points, are most likely to be reported to child protective services, and ensnared in the carceral web.

Roberts looks at affluent white neighborhoods in New York City to consider how children are cared for differently. As she describes, these neighborhoods have issues, but they are resolved without the threat of police and the carceral system, and with an assurance that needs like income, housing, and health care are met. While Roberts makes an important point about the necessity of social support, it is also important to remember that affluence and whiteness often occlude harm.^5^ This was the case for the six Black children who were adopted by Jennifer and Sara Hart, white women who abused them over many years and eventually killed them by driving their car over a cliff.^6^ As Roberts shows in her examination of this story, the ways that policing and family surveillance are absent from these neighborhoods also reinforces white supremacy, at the peril of Black families and other marginalized children.

The relevance of “Torn Apart” was clear when The Supreme Court agreed to hear challenges to the Indian Child Welfare Act of 1978 (ICWA), which was created to address the genocidal practice of removing indigenous children from their families. Weakening ICWA would have allowed the child welfare system to be used to continue the weakening indigenous communities, which are also over-criminalized.^7^ Additionally, child protective services have been weaponized against parents of trans and gender nonconforming children, with legislation to this effect in Texas and Florida.^8,9^ As Roberts warned, these are instances of the child welfare system being mobilized against groups who are oppressed because of their gender, sexuality, and ethnicity.

Among her suggestions for transforming child welfare, Roberts consistently reiterates the necessity of addressing poverty by strengthening funding and support for families. These suggestions are opportunities for researchers in public health, reproductive justice, and racial justice to engage questions that bolster the goals of advocates attempting to reduce the harms of the child welfare system. Furthermore, medical providers and other direct service providers can take lessons from this book about ways to offer care for children that do not engage the child welfare and carceral systems. Finally, “Torn Apart” should serve as a cautionary tale: those of us who seek to provide care cannot allow underlying logics of racism, sexism, classism, and homophobia to go unchallenged because we risk creating systems that reinforce those logics.

## Abbreviations Used

ICWA: Indian Child Welfare Act of 1978
PIC: prison industrial complex
